# Minigenes enhance heterologous expression and prevent aberrant splicing of mouse *Spink1*

**DOI:** 10.1038/s41598-025-13128-7

**Published:** 2025-08-02

**Authors:** Gergő Berke, Miklós Sahin-Tóth

**Affiliations:** 1 Department of Surgery, University of California Los Angeles, Los Angeles, CA 90095 USA; 2 Department of Surgery, University of California Los Angeles, 675 Charles E Young Drive South, MRL 2220, Los Angeles, CA 90095 USA

**Keywords:** Pancreatitis, Trypsin inhibitor, Adenovirus, Minigene, Pancreatic disease, Biological models

## Abstract

The digestive enzyme trypsin is an important driver of pancreatitis development and progression. Inhibition of intrapancreatic trypsin activity is an attractive yet so far unsuccessful approach for the treatment and prevention of pancreatitis. Gene therapy using adeno-associated viral vector containing the human serine protease inhibitor Kazal type 1 (*SPINK1*) gene has shown efficacy against murine pancreatitis in preclinical experiments. To advance this method to clinical testing, viral vectors with improved inhibitor expression are needed. In this study, we investigated the effect of minigenes with single introns on the expression of the mouse *Spink1* gene. We found that minigenes markedly stimulated the expression of mouse *Spink1* mRNA and SPINK1 protein in HEK 293T cells transfected with plasmids and in AR42J rat pancreatic acinar cells and isolated mouse pancreatic acini transduced with adenoviral vectors. The optimal intron size for expression enhancement was 100 nucleotides. We also demonstrated that mouse *Spink1* mRNA expressed from a cDNA construct underwent spurious mRNA splicing and minigenes prevented this splicing defect. However, enhancement of mouse *Spink1* expression by minigenes was unrelated to the rescue of aberrant mRNA splicing. The observations will facilitate the development of advanced viral vectors harboring mouse *Spink1* for preclinical therapeutic studies in mouse models of pancreatitis.

**Supplementary Information:**

The online version contains supplementary material available at 10.1038/s41598-025-13128-7.

## Introduction

The serine protease inhibitor Kazal type 1 (SPINK1), also known as the pancreatic secretory trypsin inhibitor, is a 6.2 kDa protein synthesized, stored and secreted by the pancreatic acinar cells^1,2^. SPINK1 strongly inhibits trypsin by forming a non-covalent complex in a competitive manner and thereby protects the pancreas against intrapancreatic trypsin activity^3,4^. Under physiological conditions, the digestive protease precursor trypsinogen is activated to trypsin in the small intestine, whereas ectopic trypsinogen activation inside the pancreas can lead to pancreatitis^1,2^. SPINK1 constitutes 0.1–0.8% of the total pancreatic juice protein, which corresponds to 2–13% of the trypsinogen content in molar terms^2^. Inborn loss-of-function mutations in the *SPINK1* gene increase risk for chronic pancreatitis in humans^2,5^. The mouse *Spink1* gene (legacy name *Spink3*) encodes the mouse SPINK1 protein that shares 63% sequence identity with the human inhibitor. Complete genetic deletion of *Spink1* in mice resulted in severe pancreatitis with pancreas destruction and early death^6,7^, whereas heterozygous deletion of *Spink1* accelerated the development and progression of pancreatitis in a trypsin-dependent manner^8^. A heterozygous mouse model carrying the human pathogenic *SPINK1* mutation c.194+2T>C in the mouse *Spink1* gene was shown to develop chronic pancreatitis after an experimentally induced acute attack and even spontaneously with low penetrance^9,10^. Overexpression of rat SPINK1 in mice protected against various forms of experimental pancreatitis^11–13^.

Taken together, the biochemical, human genetic and mouse modeling evidence overwhelmingly supports the notion that SPINK1 is essential for pancreas health and its protective effect is mediated via inhibition of trypsin^2^. Consequently, increasing pancreatic anti-trypsin defenses with exogenously introduced SPINK1 may be a viable option for the treatment and/or prevention of pancreatitis. Recently, using various experimental mouse models, Wang et al. (2024) demonstrated that intraperitoneal injection of recombinant adeno-associated virus carrying human *SPINK1* can effectively ameliorate pancreatitis^14^. To extend these promising preclinical experiments and eventually translate the approach to the clinical setting, improved viral vectors that enable high inhibitor expression are needed. Here, we investigated the effect of minigenes carrying single introns on the expression of mouse *Spink1* mRNA and SPINK1 protein in HEK 293T cells, AR42J rat pancreatic acinar cells and freshly isolated mouse pancreatic acini. These experiments were inspired by our previous observations that transiently transfected HEK 293T cells expressed higher levels of human SPINK1 protein from minigene constructs containing intron 1 or intron 3 in the appropriate coding DNA context^15^. More recently, we reported that a human *SPINK1* minigene construct with a 100-nucleotide mini-intron enhanced inhibitor expression^16^. This human *SPINK1* minigene was designed based on the present study with mouse *Spink1* minigenes.

## Materials & methods

### Accession number and nomenclature

NM_009258.5, *Mus musculus* serine peptidase inhibitor, Kazal type 1 (*Spink1*), mRNA. The legacy name of the mouse *Spink1* gene is *Spink3*, which was used in former publications. According to convention, we denote the human gene as *SPINK1* and the mouse gene as *Spink1*, while the protein is indicated as SPINK1 for both species.

### Plasmid constructs

Construction of the pcDNA3.1(-) plasmid harboring the coding DNA (cDNA) for the mouse SPINK1 protein with a C-terminal 10His tag and the minigene 1892 construct was described previously^17^. All other mouse *Spink1* gene constructs were generated by gene synthesis (GenScript, Piscataway, NJ) and cloned into the pcDNA3.1(-) vector using the XhoI – HindIII (10His-tagged minigenes) or XhoI - BamHI (untagged constructs) sites. The 10His-tagged *Spink1* constructs contained a shortened 3’ UTR (57 nucleotides) without the native polyadenylation signal. In the untagged *Spink1* constructs, the 3’ UTR was restored to 91 nucleotides including the polyadenylation signal, based on the NM_009258.5 reference sequence (Figure S1).

### Adenovirus constructs

The 10His-tagged *Spink1* cDNA and minigene 100 gene constructs were transferred from the pcDNA3.1(-) plasmid to the VQAd5CMVK-NpA shuttle vector using the XhoI and BamHI restriction sites. Replication-defective, recombinant adenovirus was generated by Viraquest (North Liberty, IA). The titrated concentration of the *Spink1* cDNA adenoviral stock solution was 3 × 10^10^ plaque-forming unit (pfu) per mL. The *Spink1* minigene 100 adenovirus could not be reliably titrated because it formed plaques poorly. Therefore, the virus concentration was estimated from the viral particle concentration as 2.5 × 10^10^ pfu per mL.

### Cell culture and transfection/transduction

Cell culture reagents were purchased from Thermo Fisher Scientific. HEK 293T cells (catalog number Q401, GenHunter, Nashville, TN) were maintained in Dulbecco’s Modified Eagle Medium (DMEM, catalog number 10313039) supplemented with 10% fetal bovine serum (catalog number 16000044), 4 mM L-glutamine (catalog number 25030081), and 100 U/mL penicillin, 100 µg/mL streptomycin (catalog number 15140122) at 37 °C. The cells were seeded in 6-well tissue culture plates at 1.5 × 10^6^ cells per well density. Transfections were carried out by adding 0.5 mL Opti-MEM medium (catalog number 11058021) containing 4 µg plasmid DNA and 5 µL Lipofectamine 2000 (catalog number 11668019) to 1.5 mL DMEM medium. Cells were incubated overnight with the transfection mix, rinsed with 1 mL phosphate-buffered saline (pH 7.4), and supplemented with 1.5 mL Opti-MEM. Conditioned media and cells were harvested for analysis 48 h after the addition of the Opti-MEM medium.

AR42J rat pancreatic acinar cells (catalog number #CRL-1492, ATCC) were grown in DMEM supplemented with 20% fetal bovine serum, 4 mM glutamine, 100 U/mL penicillin, and 100 µg/mL streptomycin at 37 °C. The cells were seeded in 6-well plates at a density of 10^6^ cells per well, treated with 100 nM dexamethasone for 48 h, and rinsed twice with 1 mL phosphate-buffered saline (pH 7.4). Recombinant adenovirus was diluted with Opti-MEM and transduction was performed with 1 mL virus solution containing to the indicated viral dose and 100 nM dexamethasone. Conditioned media and cells were harvested 24 h after the addition of the adenovirus.

### Isolation of mouse pancreatic acinar cells

Pancreatic acini were isolated from 10-week-old C57BL/6N female mice (purchased from Charles River Laboratories, Wilmington, MA) by collagenase digestion (collagenase type 4, 0.22 μm filtered, code CLSS-4, catalog #LS004209, Worthington Biochemical, Lakewood NJ) according to published protocols^18–21^. Mice were euthanized with carbon dioxide followed by cervical dislocation as secondary method. Pancreata from 2 mice were pooled for the isolation procedure and two independent preparations were made. After the last washing step, the cells were suspended in 10 mL incubation solution (DMEM/F-12 medium with 1 mg/mL bovine serum albumin), seeded in 48-well tissue culture plates (500 µL per well) and allowed to recover for 30 min before adding the indicated adenoviral dose in 500 µL incubation solution. Conditioned medium and cells were collected 24 h after transduction. Because soybean trypsin inhibitor interfered with the measurement of SPINK1 protein by active-site titration, it was omitted from the incubation solution.

### Animal study approval

All experiments were performed in accordance with relevant guidelines and regulations. Animal experiments were conducted and reported in accordance with the Animal Research: Reporting of In Vivo Experiments (ARRIVE) guidelines. Studies were carried out at the University of California, Los Angeles (UCLA) with the approval and oversight of the Animal Research Committee, including protocol review and post-approval monitoring. The animal care program at UCLA is managed in full compliance with the US Animal Welfare Act, the United States Department of Agriculture Animal Welfare Regulations, the US Public Health Service Policy on Humane Care and Use of Laboratory Animals and the National Research Council’s Guide for the Care and Use of Laboratory Animals. UCLA has an approved Animal Welfare Assurance statement on file with the US Public Health Service, National Institutes of Health, Office of Laboratory Animal Welfare, and it is accredited by the Association for Assessment and Accreditation of Laboratory Animal Care International (AAALAC).

### Protein gel electrophoresis

Aliquots of the conditioned media (175 µL) were precipitated with trichloroacetic acid (20% final concentration) and centrifuged for 10 min at 16,000*g*, 4 °C. The protein pellet was resuspended in 25 µL 2× Laemmli sample buffer (catalog number 1610737, Bio-Rad, Hercules, CA) supplemented with 100 mM dithiothreitol and 150 mM NaOH. The samples were heat denatured at 95 °C for 20 min, electrophoresed on 15% SDS polyacrylamide minigels, and stained with Brilliant Blue R-250 (Coomassie Blue).

### Active-site Titration

Secreted SPINK1 protein levels in the conditioned medium were measured by titration against mouse cationic trypsin. The concentration of cationic trypsin was determined by titration against the pan-protease inhibitor ecotin. Briefly, 10–50 µL of conditioned medium was diluted to 100 uL with assay buffer (0.1 M Tris-HCl (pH 8.0), 1 mM CaCl_2_, and 0.05% Tween 20) and a 2-fold serial dilution was prepared in a microplate (50 µL final volume per well). A 50 µL aliquot of a 20 nM cationic trypsin solution was added to each well and the 100 µL mixture was incubated at 22 °C for 30 min. The residual trypsin activity was measured with 100 µL of 200 µM Z-Gly-Pro-Arg-*p*-nitroanilide substrate (dissolved in assay buffer). Residual trypsin activity was plotted as a function of the conditioned medium volume in each well, and the linear portion of the inhibition curve was extrapolated to the *x* intercept to determine the volume of conditioned medium that fully inhibited the added trypsin. The SPINK1 concentration was then calculated using the C_1_V_1_ = C_2_V_2_ dilution equation.

### RNA isolation and reverse-transcription PCR

Total RNA was isolated from HEK 293T cells, AR42J cells and mouse pancreatic acini using the RNeasy Plus Mini Kit (Qiagen, Valencia CA). RNA (2 µg) was reverse-transcribed with the High Capacity cDNA Reverse Transcription Kit (catalog number 4368814, Thermo Fisher Scientific). Splicing of mouse *Spink1* mRNA was analyzed by amplifying the cDNA with the following primers. Forward: 5’- CCA GAT CTT CGA CAA TGA AGG − 3’, reverse: 5’- CGG TAG CCA TAA CAG AGT TC −3’ (Figure S1). The PCR products were resolved on 2% agarose gels and stained with GreenGlo Safe DNA Dye (catalog number C788T73, Thomas Scientific, Swedesboro, NJ). Quantitative PCR was performed with the Taqman Universal PCR Master Mix (catalog number: 4304437, Thermo Fisher Scientific) and the Mm00436765_m1 (mouse *Spink1*), Hs02758991_g1 (human *GAPDH*, used for HEK 293T cells), Rn01775763_g1 (rat *Gapdh*, used for AR42J cells), and Mm01612987_g1 (mouse *Rpl13a*, used for mouse acini) TaqMan probes. Relative expression levels were calculated with the ΔΔCT method.

## Results

### Intron-mediated enhancement of mouse *Spink1* expression

**Fig. 1:**
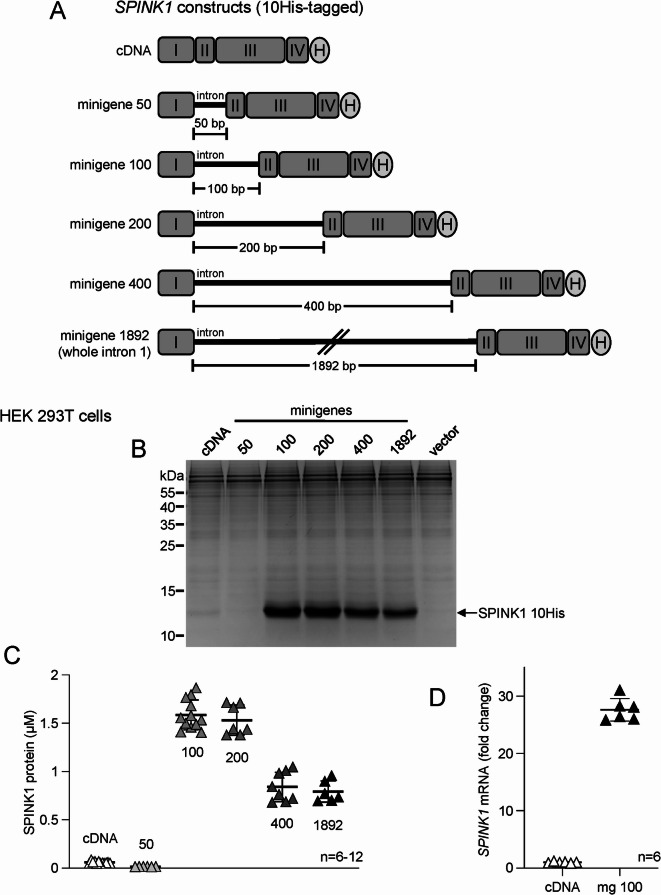
Intron-mediated enhancement of expression of mouse *Spink1* in HEK 293T cells. (**A**) Schematic representation of 10His-tagged (H) expression constructs used for transfections with mouse *Spink1* cDNA or the indicated minigenes. Exons numbered with Roman numerals are illustrated by rounded rectangles and the introns are indicated by the horizontal lines. (**B**) Levels of secreted mouse SPINK1 protein in the conditioned medium visualized by SDS-PAGE and Coomassie Blue staining. Representative gel is shown. Note the strong staining of the 10His-tagged mouse SPINK1 protein (compare with Fig. 4C). **(C)** Mouse SPINK1 protein levels in the conditioned medium determined by active-site titration against mouse cationic trypsin. Individual values from 3–6 transfections with duplicates (*n* = 6–12) are shown, with the mean and SD indicated. (**D)** Expression of mouse *Spink1* mRNA in cells transfected with the cDNA and minigene 100 constructs. *Spink1* mRNA levels were measured by reverse-transcription quantitative PCR and expressed as fold change relative to the average value of the cDNA construct. Individual values from 3 transfections with duplicates (*n* = 6) are shown, with the mean and SD indicated.

To boost heterologous expression of mouse *Spink1* from plasmid and viral vectors via intron-mediated enhancement, we placed the 1892 nucleotide long intron 1 from the human *SPINK1* gene in the mouse coding sequence between exons 1 and 2 (Fig. 1A)^17^. To test the effect of intron size, we generated mouse *Spink1* minigenes with introns truncated to 400, 200, 100, and 50 nucleotides by deleting sequences from the middle portion and preserving the ends in a symmetrical fashion. All constructs contained a C-terminal polyhistidine (10His) affinity tag and a shortened 3’ UTR without the native polyadenylation site (Figure S1). Mouse SPINK1 with a C-terminal 10His tag is a fully functional inhibitor, which has been used in previous studies^4,17^.

First, we transfected HEK 293T cells with the expression plasmids and measured the secreted mouse SPINK1 protein from the conditioned medium using SDS-PAGE with Coomassie Blue staining (Fig. 1B, Figure S2) and active-site titration against mouse cationic trypsin (Fig. 1C). Relative to the coding DNA (cDNA) construct, minigenes 1892, 400, 200, and 100 showed 13.6-fold, 14.1-fold, 26.2-fold, and 27.1-fold enhanced expression of mouse SPINK1 protein, respectively. In contrast, no SPINK1 protein was secreted by cells transfected with minigene 50. Thus, the optimal intron size for enhanced mouse *Spink1* expression was 100–200 nucleotides while larger introns resulted in less efficient enhancement and a shorter intron was completely ineffective. To confirm that the increased secretion of SPINK1 protein was due to higher mRNA expression, we used reverse-transcription quantitative PCR to compare mouse *Spink1* mRNA levels in cells with cDNA and minigene 100 constructs (Fig. 1D). Remarkably, transfection with minigene 100 versus cDNA constructs resulted in 27.6-fold higher *Spink1* mRNA expression.

**Fig. 2:**
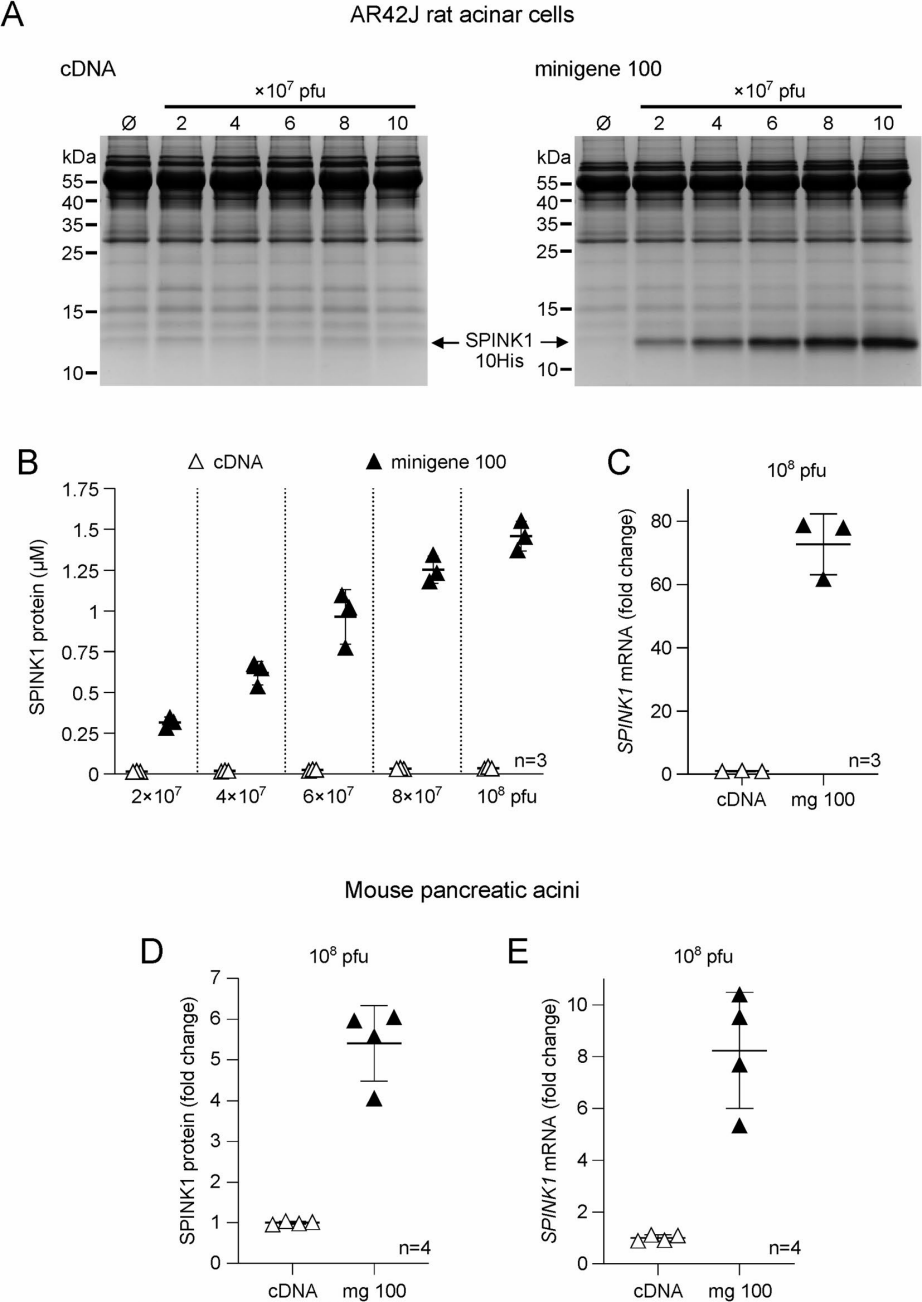
Intron-mediated enhancement of expression of mouse *Spink1* in AR42J and mouse pancreatic acinar cells. The rat pancreatic acinar cell line and freshly isolated mouse pancreatic acini were transduced with recombinant adenovirus carrying 10His-tagged mouse *Spink1* cDNA or minigene 100 constructs. The adenovirus doses used for transductions are indicated in plaque-forming units (pfu). (**A**) Levels of secreted mouse SPINK1 protein in the conditioned medium of AR42J cells were visualized by SDS-PAGE and Coomassie Blue staining. Representative gels are shown. (**B)** Mouse SPINK1 protein levels in the conditioned medium of AR42J cells were determined by active-site titration against mouse cationic trypsin. Individual values from 3 transductions are shown, with the mean and SD indicated. (**C)** Expression of mouse *Spink1* mRNA in AR42J cells. *Spink1* mRNA levels were measured by reverse-transcription quantitative PCR and expressed as fold change relative to the average value of the cDNA construct within each transduction. Individual values from 3 transductions are shown, with the mean and SD indicated. (**D)** Mouse SPINK1 protein levels in the conditioned medium of mouse pancreatic acini measured by active-site titration against mouse cationic trypsin. Individual values from 2 transductions in duplicates using 2 independent acinar cell preparations are shown (*n* = 4), with the mean and SD indicated. Note that mouse pancreatic acini secrete endogenous SPNK1 protein and exhibit higher variability than established cell lines. Therefore, results were expressed as fold change relative to the cDNA constructs. (**E**) Expression of mouse *Spink1* mRNA in transduced mouse pancreatic acini. *Spink1* mRNA levels were measured by reverse-transcription quantitative PCR and expressed as fold change relative to the average value of the cDNA construct within each transduction. Individual values from 2 transductions in duplicates using 2 independent acinar cell preparations are shown (*n* = 4), with the mean and SD indicated. Note that mouse pancreatic acini also express endogenous *Spink1* mRNA, which is measured together with the transduced gene products.

To demonstrate that minigenes could enhance mouse *Spink1* expression in pancreatic acinar cells, we generated recombinant adenoviral vectors with the 10His-tagged cDNA and minigene 100 constructs and transduced AR42J cells. This widely used cell line originated from azaserine-induced rat pancreatic acinar cell tumors^22,23^. Cells were treated with dexamethasone to upregulate the secretory pathway^24,25^ and then transduced with viruses carrying *Spink1* cDNA or minigene 100 constructs using doses ranging from 2 × 10^7^ to 10^8^ plaque-forming units (pfu). Secreted mouse SPINK1 protein was measured from the conditioned medium by SDS-PAGE (Fig. 2A, Figure S2) and active-site titration (Fig. 2B). SPINK1 protein levels increased in the medium as a linear function of the viral dose employed. AR42J cells transduced with the minigene 100 construct secreted significantly higher levels of SPINK1 protein than those transduced with cDNA. The average expression difference between the two constructs across the 5 virus doses employed was 34.5 ± 8-fold (mean ± SD). As seen with HEK 293T cells, the enhanced SPINK1 protein secretion was due to changes in *Spink1* mRNA expression, which was 72.7-fold elevated in cells with minigene 100 versus those with cDNA at the 10^8^ viral dose (Fig. 2C). Even though AR42J cells secrete various pancreatic enzymes, we could not detect endogenous trypsin inhibitory activity in the conditioned medium, suggesting that none of the two rat pancreatic SPINK isoforms (SPINK1 and SPINK3) are secreted.

Finally, we freshly isolated mouse acini from the pancreas of C57BL/6N mice and transduced the cells with a 10^8^ pfu dose of adenovirus carrying mouse *Spink1* cDNA or minigene 100 constructs. Due to lower expression levels and the presence of bovine serum albumin in the medium, we were unable to visualize the secreted SPINK1 protein on Coomassie Blue-stained SDS-PAGE gels. Active-site titration revealed 5.4-fold increased SPINK1 protein levels in the medium from cells expressing minigene 100 compared to those with cDNA (Fig. 2D). In agreement with the protein levels, reverse-transcription quantitative PCR indicated 8.2-fold increased mRNA expression in cells transduced with minigene 100 relative to those with cDNA (Fig. 2E). Compared to HEK 293T and AR42J cells, the seemingly smaller enhancement of expression by minigene 100 in mouse pancreatic acini is explained by the expression of endogenous mouse *Spink1* mRNA and SPINK1 protein, which are measured together with the exogenously expressed forms and confound the calculation of their true ratio.

### Minigene 100 rescues spurious mRNA splicing of mouse *Spink1* cDNA

**Fig. 3:**
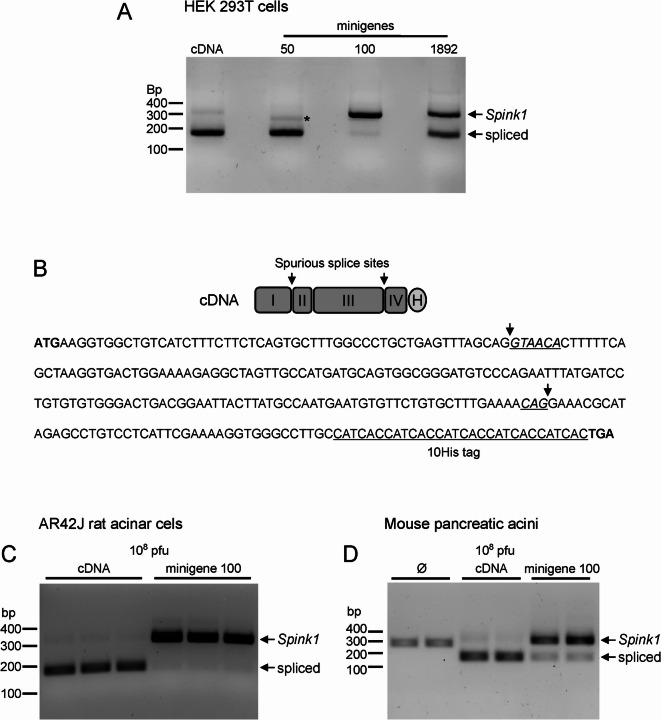
Aberrant splicing of mouse *Spink1* cDNA and rescue by minigenes. (**A**) Splicing of mouse *Spink1* mRNA in HEK 293T cells expressed from cDNA and indicated minigene constructs was analyzed by reverse-transcription PCR and agarose gel electrophoresis. The spurious splicing joins nucleotide c.55 to c.198, resulting in the deletion of 142 nucleotides. The faint band in the minigene 50 sample indicated by the asterisk corresponds to an aberrant splice product in which nucleotide c.6 is spliced to c.56 resulting in the deletion of 49 nucleotides. (**B)** Schematic representation and nucleotide sequence of the mouse *Spink1* cDNA. The spurious splice sites are italicized and underlined. (**C)** Splicing of mouse *Spink1* mRNA in AR42J cells transduced with *Spink1* cDNA and minigene 100 constructs. (**D)** Splicing of mouse *Spink1* mRNA in mouse pancreatic acini transduced with *Spink1* cDNA and minigene 100 constructs. Note that untransduced cells (Ø) express endogenous *Spink1* mRNA that is not subject to spurious splicing. When transduced with adenoviral constructs, the endogenous band is not amplified to a visible degree due to competition with the exogenous *Spink1* mRNA.

We used reverse-transcription PCR and agarose gel electrophoresis to visualize the splicing efficiency of mouse *Spink1* minigenes 1892, 100, and 50 (Fig. 3A, Figure S3). The cDNA construct served as the normal size control. Unexpectedly, we found that the mRNA expressed from the *Spink1* cDNA underwent spurious splicing that resulted in a smaller than expected PCR product. DNA sequencing revealed that the aberrant splicing excised 142 nucleotides from the *Spink1* mRNA. The shortened message would code for a functionally defective inhibitor protein. The mRNA splicing took place at canonical sites, which were accidentally generated at the exon junctions in the cDNA (Fig. 3B). Minigene 100 almost completely prevented spurious splicing while minigene 1892 was partly effective and minigene 50 provided no rescue. The aberrant splicing of mouse *Spink1* cDNA and rescue by minigene 100 was also confirmed in AR42J cells (Fig. 3C, Figure S3) and in mouse pancreatic acini (Fig. 3D, Figure S3). DNA sequencing of the PCR amplicon of the *Spink1* mRNA expressed from the minigene 100 construct confirmed the correct splicing of the intron.

### Enhancement of mouse *Spink1* expression is not related to rescue of aberrant splicing

**Fig. 4:**
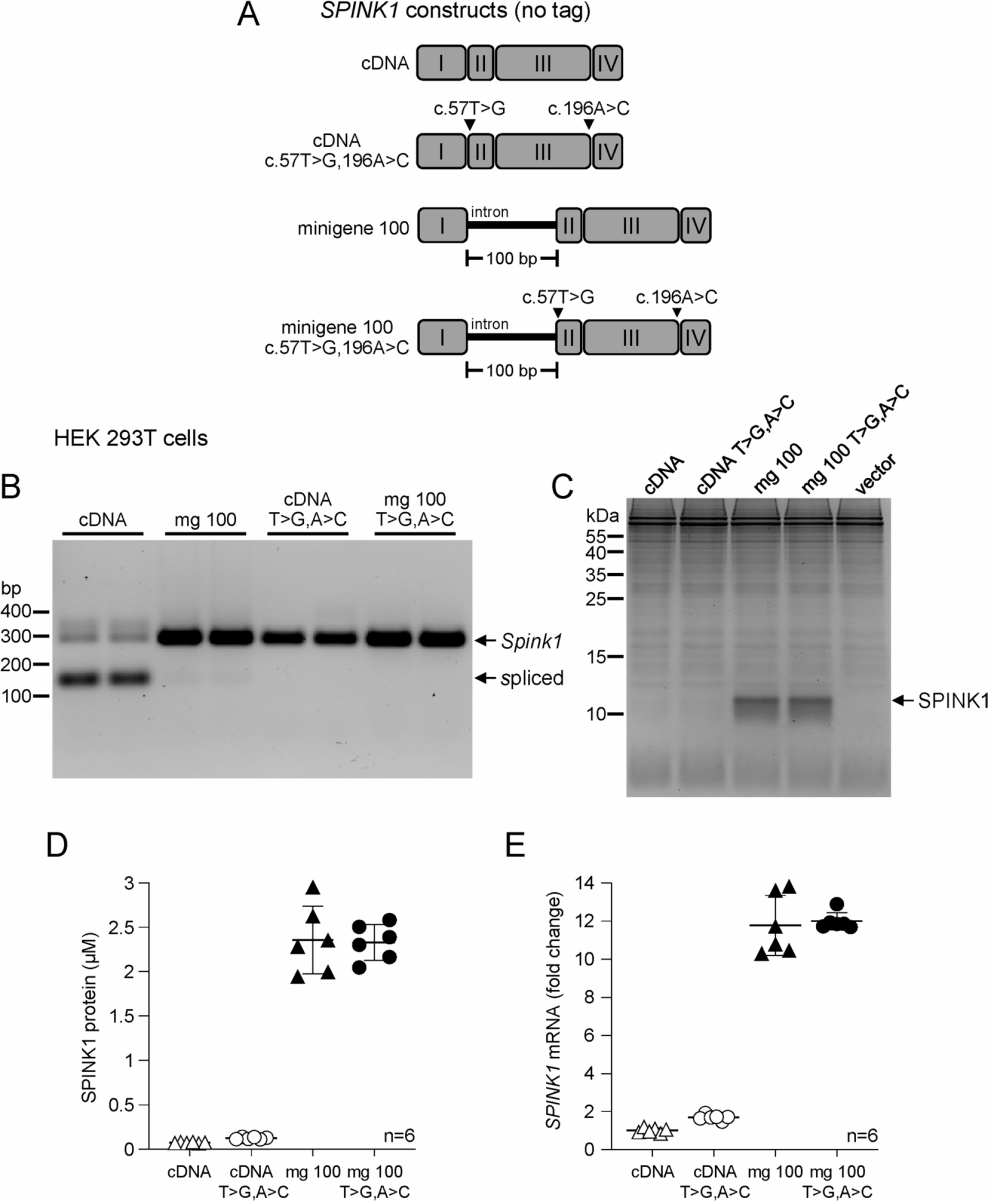
Rescue of aberrant splicing of mouse *Spink1* mRNA in HEK 293T cells by mutation of spurious splice sites or minigene 100. (**A**) Schematic representation of untagged expression constructs used for transfections. Exons numbered with Roman numerals are illustrated by rounded rectangles and the introns are represented by the horizontal lines. Silent mutations that eliminate the aberrant splice sites are indicated. (**B)** Splicing of mouse *Spink1* mRNA in cells transfected with *Spink1* cDNA and minigene 100 constructs without or with the splice-site mutations. (**C)** Levels of secreted mouse SPINK1 protein in the conditioned medium visualized by SDS-PAGE and Coomassie Blue staining. Representative gel is shown. Note the weaker staining of the untagged mouse SPINK1 protein (compare with Fig. 1B). **(D)** Mouse SPINK1 protein levels in the conditioned medium determined by active-site titration against mouse cationic trypsin. Individual values from 3 transfections with duplicates (*n* = 6) are shown, with the mean and SD indicated. (**E)** Expression of mouse *Spink1* mRNA in cells transfected with the cDNA and minigene 100 constructs without or with the splice-site mutations. *Spink1* mRNA levels were measured by reverse-transcription quantitative PCR and expressed as fold change relative to the average value of the cDNA construct within each transfection. Individual values from 3 transfections with duplicates (*n* = 6) are shown, with the mean and SD indicated.

The observation that the minigene 100 construct prevents the spurious mRNA splicing of *Spink1* cDNA suggests that enhancement of expression might be due to this effect. To test this notion, we eliminated the aberrant splicing of mouse *Spink1* cDNA by mutating both splice sites using silent mutations c.57T>G (p.Gly19=) and c.196A>C (p.Arg66=). As a control, we introduced the same mutations in the minigene 100 construct as well. For these experiments, we generated 4 new *Spink1* plasmid constructs (Fig. 4A) without the C-terminal 10His tag (cDNA, minigene 100, cDNA with c.57T>G,196A>C and minigene 100 with c.57T>G,196A>C), to rule out the unlikely possibility that the tag sequence somehow alters mRNA splicing or contributes to expression enhancement. In these new constructs, we also restored the full length 3’ UTR sequence together with the native polyadenylation signal (Figure S1). HEK 293T cells were transfected and reverse-transcription PCR with agarose gel analysis was used to analyze mRNA splicing. The results confirmed that the aberrant splicing was completely rescued by minigene 100 or the splice-site mutations (Fig. 4B, Figure S4). Surprisingly, however, SDS-PAGE with Coomassie Blue staining (Fig. 4C, Figure S4), active-site titration (Fig. 4D) and reverse-transcription quantitative PCR (Fig. 4E) consistently demonstrated that rescue of aberrant splicing by the splice-site mutations increased expression only 1.7-fold, whereas minigene 100, without or with the splice-site mutations, enhanced expression 31-fold and 30.6-fold (SPINK1 protein) and 11.8-fold and 12-fold (*Spink1* mRNA), respectively. Interestingly, compared to the 10His-tagged constructs, the untagged constructs with the restored native 3’ UTR yielded 1.5-fold higher levels of secreted SPINK1 protein in the medium. Taken together, the observations indicate that enhancement of mouse *Spink1* expression by the minigene 100 construct is unrelated to the rescue of aberrant mRNA splicing.

## Discussion

Intron-mediated enhancement of gene expression is an evolutionarily conserved phenomenon that has been described in a variety of eukaryotes^26,27^. Introns and their removal via splicing generally has a positive effect on gene expression, which may be related to improved transcription, more efficient RNA export, and slower mRNA decay or a combination of these processes^28,29^. Intron-dependent gene-looping can facilitate direct interactions between the promoter and terminator regions as well as the intronic splice sites^30^. Exon-junction complexes enhance expression by suppressing spurious splice sites and preventing unwanted re-splicing of mRNA^31–33^. Exon-junction complexes can also increase translation efficiency^34^.

In the present study, we investigated the intron-mediated enhancement of expression of the mouse pancreatic trypsin inhibitor gene *Spink1*. Our goal was to boost expression of mouse *Spink1* from viral vectors for the purpose of therapeutic trypsin inhibition in mouse models of pancreatitis. Recent preclinical studies in murine pancreatitis using an adeno-associated viral vector harboring the human *SPINK1* cDNA offered proof of concept that gene therapy may be utilized to enhance pancreatic defenses against unwanted intrapancreatic trypsin activity and pancreatitis^14^. In our experiments, we demonstrated that minigenes containing an intron from human *SPINK1* could be used to enhance expression of mouse *Spink1* mRNA and SPINK1 protein. Furthermore, we found that the smallest optimal intron size for expression enhancement is 100 nucleotides (minigene 100) and proved the general utility of the approach in the AR42J rat pancreatic acinar cell line and in freshly isolated mouse pancreatic acinar cells using adenoviral vectors. Finally, we showed that mouse *Spink1* mRNA expressed from a cDNA construct underwent spurious splicing and minigene 100 rescued this aberrant reaction. Enhancement of *Spink1* expression by the minigene 100 construct, however, was largely unrelated to the correction of spurious mRNA splicing.

The empirically determined smallest yet optimal size of an intron for the purpose of expression enhancement was 100 nucleotides. Increasing intron size to 200 nucleotides had minimal impact while further extension to 400 or 1892 nucleotides decreased the efficiency of expression enhancement. While an explanation for this observation is not obvious, we speculate that steric factors associated with increased intron size might play a role. Interestingly, however, there was no significant difference between the enhancing effect of introns with 400 and 1892 nucleotides, which may suggest a size threshold. Reducing intron size to 50 nucleotides caused no enhancement whatsoever and resulted in diminished mouse SPINK1 protein expression. The reason for the splicing failure is not readily apparent, because the 50-nucleotide intron still contained the essential elements required for the splicing reaction, i.e. the 5’ splice site, the branch point sequence, the polypyrimidine tract, and the 3’ splice site^35^. We note, however, that the minimum eukaryotic intron size is approximately 30–40 nucleotides^36^, indicating that a small intron may not be compatible with efficient splicing.

An unexpected and novel observation of this study is the aberrant splicing of the mouse *Spink1* mRNA when expressed from a cDNA construct. The splicing occurred at canonical splice sites that seemed to have formed fortuitously at the exon connections within the cDNA. This is not unusual, however, since the 3’ end of exons frequently contains the CAG sequence, which corresponds to the consensus of the 3’ (acceptor) splice site. Similarly, the 5’ end of exons often starts with the GT nucleotides, the consensus sequence for the 5’ (donor) splice site. Thus, within any cDNA sequence potential splice sites may be present, which, given the right arrangement and the presence of a branchpoint sequence and a polypyrimidine tract, may result in spurious splicing of the cDNA construct when expressed heterologously. Note that this problem does not exist during physiological gene expression as the presence of introns suppresses any aberrant splicing^32,33^, as also seen with the minigene 100 construct in our experiments. Since cDNA constructs are routinely used for heterologous expression of genes, spurious mRNA splicing might be a reason for unsuccessful experiments and should be considered during troubleshooting. Interestingly, when we eliminated unwanted splicing by mutating the splice sites within the cDNA, mouse *Spink1* expression levels were increased only 1.7-fold, indicating that intron-mediated enhancement of mouse *Spink1* expression is largely independent of the prevention of aberrant splicing. This notion is further supported by the fact that expression of human *SPINK1* is also strongly enhanced by the minigene 100 construct even though the human cDNA does not suffer aberrant mRNA splicing^16^.

 1In summary, we demonstrated that the use of a minigene construct harboring a short intron markedly stimulated expression of mouse *Spink1* mRNA and SPINK1 protein. The enhancement effect was equally impressive in HEK 293T cells transfected with plasmids and in AR42J rat pancreatic acinar cells and mouse pancreatic acini transduced with adenoviral vectors. The findings will facilitate the development of improved viral vectors harboring mouse *Spink1* for therapeutic trypsin inhibition in mouse models of pancreatitis. Further research is needed to determine whether minigene-mediated enhancement is a generally applicable method in heterologous gene expression systems, or its utility may be target-gene dependent.

## Supplementary Information

Below is the link to the electronic supplementary material.

Supplementary Material 1

## Data Availability

All data generated or analyzed during this study are included in this published article and its supplementary information file.
