# Supplementary material for: Minigenes enhance heterologous expression and prevent aberrant splicing of mouse Spink1
Source: Sci Rep. 2025 Aug 2;15:28222. doi: 10.1038/s41598-025-13128-7 (PMC12318039; doi:10.1038/s41598-025-13128-7)
Supplement: Supplementary file 1 — Supplementary Material 1 [file 41598_2025_13128_MOESM1_ESM.pdf]

Supplementary Figure S1. Mouse *Spink1* cDNA and minigene 100 constructs used in this study. The translation start and stop codons are emboldened and highlighted in green. The 10His tag is in gray. The intron is shown in blue and the splice sites are italicized and underlined. The polyadenylation signal is emboldened and underlined. The primers used for reverse-transcription PCR are also indicated.

Mouse *Spink1* 10His cDNA construct

XhoI
CTCGAGTTCTTCTGGCTTTTGCAC**CCAGATCTTCGACAATGAAGG**TGGCTGTCATCTTTCTTCTCAGTGCTTTGGCC
CTGCTGAGTTTAGCAGGTAACACTTTTTCAGCTAAGGTGACTGGAAAAGAGGCTAGTTGCCATGATGCAGTGGCGGG
ATGTCCCAGAATTTATGATCCTGTGTGTGGGACTGACGGAATTACTTATGCCAATGAATGTGTTCTGTGCTTTGAAA
ACAGGAAACGCATAGAGCCTGTCCTCATTCGAAAAGGTGGGCCTTGCCATCACCATCACCATCACCATCACCATCAC
TGAAAGTCAAGATTTT**GAACTCTGTTATGGCTACCG**TAATGTTTGGCAAGTGGGTTCGTTGGATCC
BamHI

Mouse *Spink1* 10His minigene 100 construct

XhoI
CTCGAGTTCTTCTGGCTTTTGCA**CCCAGATCTTCGACAATGAAGG**TGGCTGTCATCTTTCTTCTCAGTGCTTTGGCC
CTGCTGAGTTTAGCAG*GTAAGT*GTTGCATATTTTTCAAATTTAAATAAAACTGTTTTGACCTGTTGCTGACTTCTTT
CATTTAGGACCCAACTTACCATATCTGATTTATTTC*TAG*GTAACACTTTTTCAGCTAAGGTGACTGGAAAAGAGGCT
AGTTGCCATGATGCAGTGGCGGGATGTCCCAGAATTTATGATCCTGTGTGTGGGACTGACGGAATTACTTATGCCAA
TGAATGTGTTCTGTGCTTTGAAAACAGGAAACGCATAGAGCCTGTCCTCATTCGAAAAGGTGGGCCTTGCCATCACC
ATCACCATCACCATCACCATCAC**TGA**AAGTCAAGATTTT**GAACTCTGTTATGGCTACCG**TAATGTTTGGCAAGTGGG
TTCGTTAAGCTT
HindIII

Mouse *Spink1* untagged cDNA construct

XhoI
CTCGAGTTCTTCTGGCTTTTGCAC**CCAGATCTTCGACAATGAAGG**TGGCTGTCATCTTTCTTCTCAGTGCTTTGGCC
CTGCTGAGTTTAGCAGGTAACACTTTTTCAGCTAAGGTGACTGGAAAAGAGGCTAGTTGCCATGATGCAGTGGCGGG
ATGTCCCAGAATTTATGATCCTGTGTGTGGGACTGACGGAATTACTTATGCCAATGAATGTGTTCTGTGCTTTGAAA
ACAGGAAACGCATAGAGCCTGTCCTCATTCGAAAAGGTGGGCCTTGC**TGA**AAGTCAAGATTTT**GAACTCTGTTATGG**
CTACCGTAATGTTTGGCAAGTGGGTTCGTTG**AATAAA**TGCATCTGAACATACCTTGTTCTTCCCGGATCC
BamHI

Mouse *Spink1* untagged minigene100 construct

XhoI
CTCGAGTTCTTCTGGCTTTTGCAC**CCAGATCTTCGACAATGAAGG**TGGCTGTCATCTTTCTTCTCAGTGCTTTGGCC
CTGCTGAGTTTAGCAG*GTAAGT*GTTGCATATTTTTCAAATTTAAATAAAACTGTTTTGACCTGTTGCTGACTTCTTT
CATTTAGGACCCAACTTACCATATCTGATTTATTTC*TAG*GTAACACTTTTTCAGCTAAGGTGACTGGAAAAGAGGCT
AGTTGCCATGATGCAGTGGCGGGATGTCCCAGAATTTATGATCCTGTGTGTGGGACTGACGGAATTACTTATGCCAA
TGAATGTGTTCTGTGCTTTGAAAACAGGAAACGCATAGAGCCTGTCCTCATTCGAAAAGGTGGGCCTTGC**TGA**AAGT
CAAGATTTT**GAACTCTGTTATGGCTACCG**TAATGTTTGGCAAGTGGGTTCGTTG**AATAAA**TGCATCTGAACATACCT
TGTTCTTCCCGGATCC
BamHI

Figure 1B uncropped protein gel picture

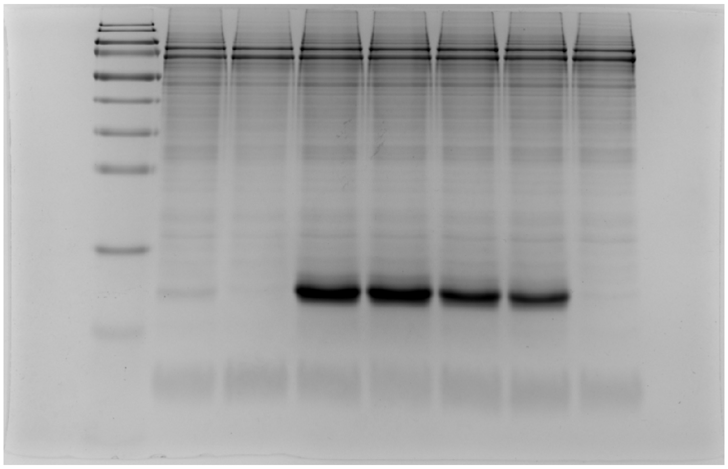

Figure 2A uncropped protein gel pictures

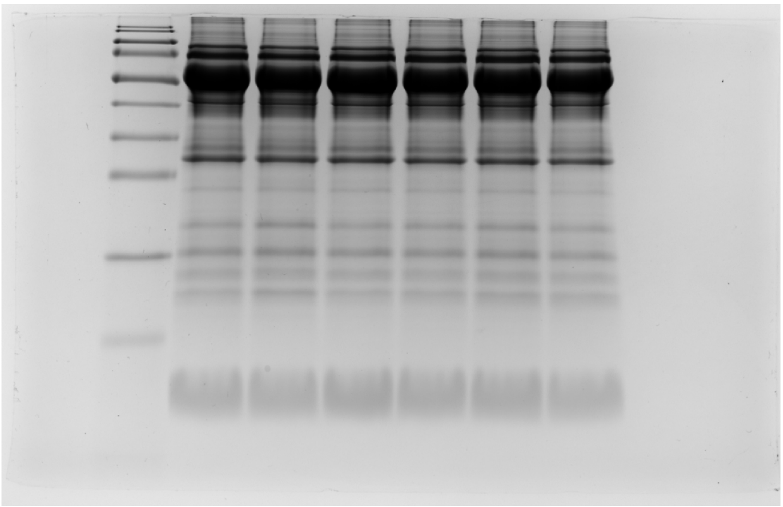

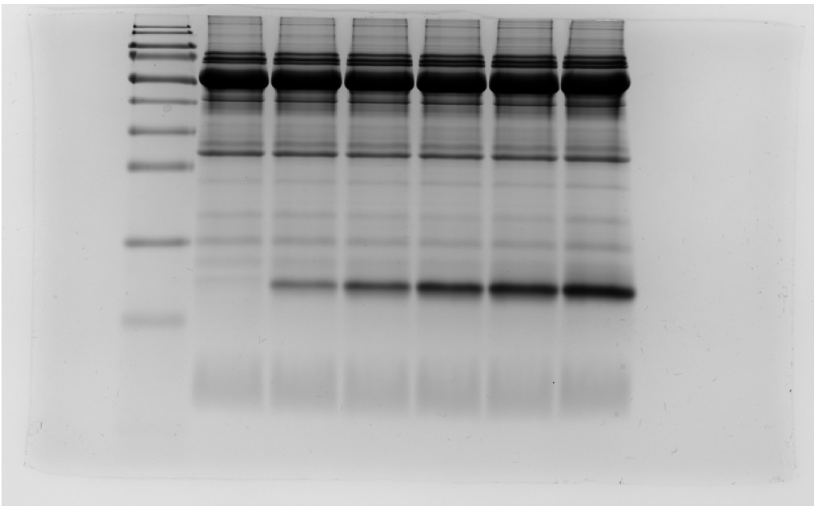

Supplementary Figure S2

Figure 3A and 3C uncropped agarose gel pictures

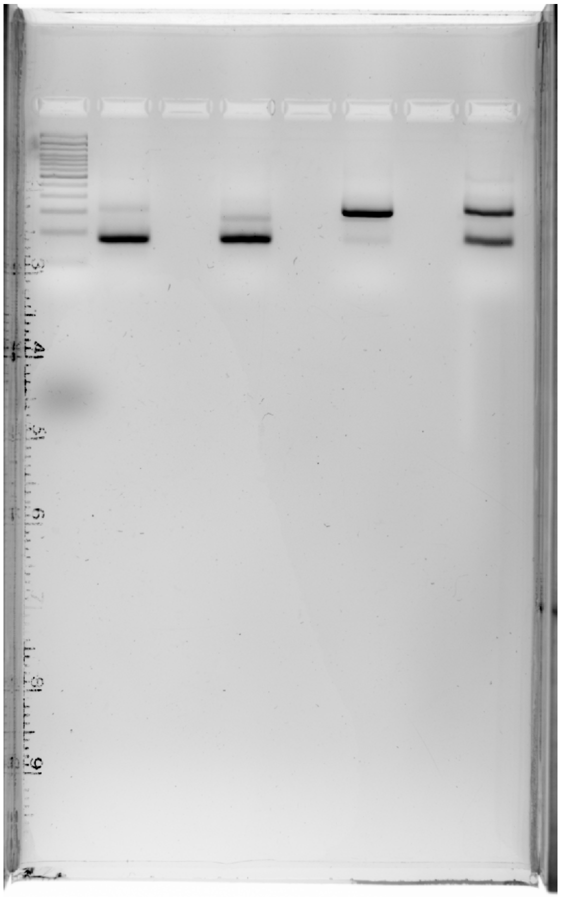

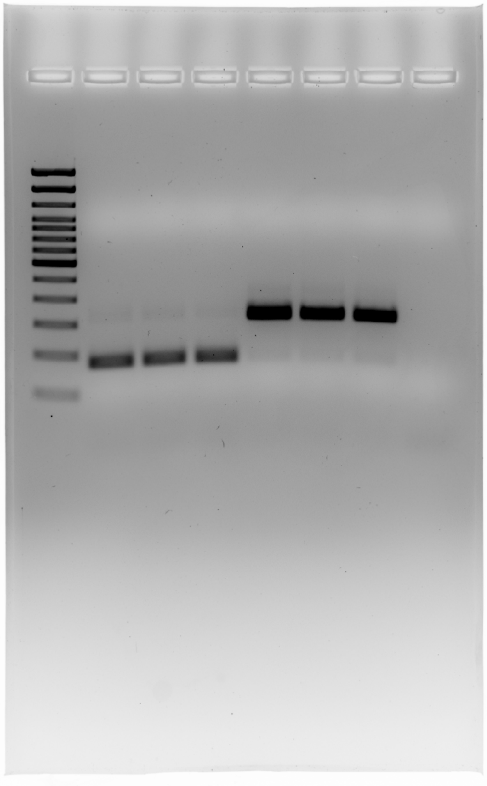

Figure 3D uncropped agarose gel picture

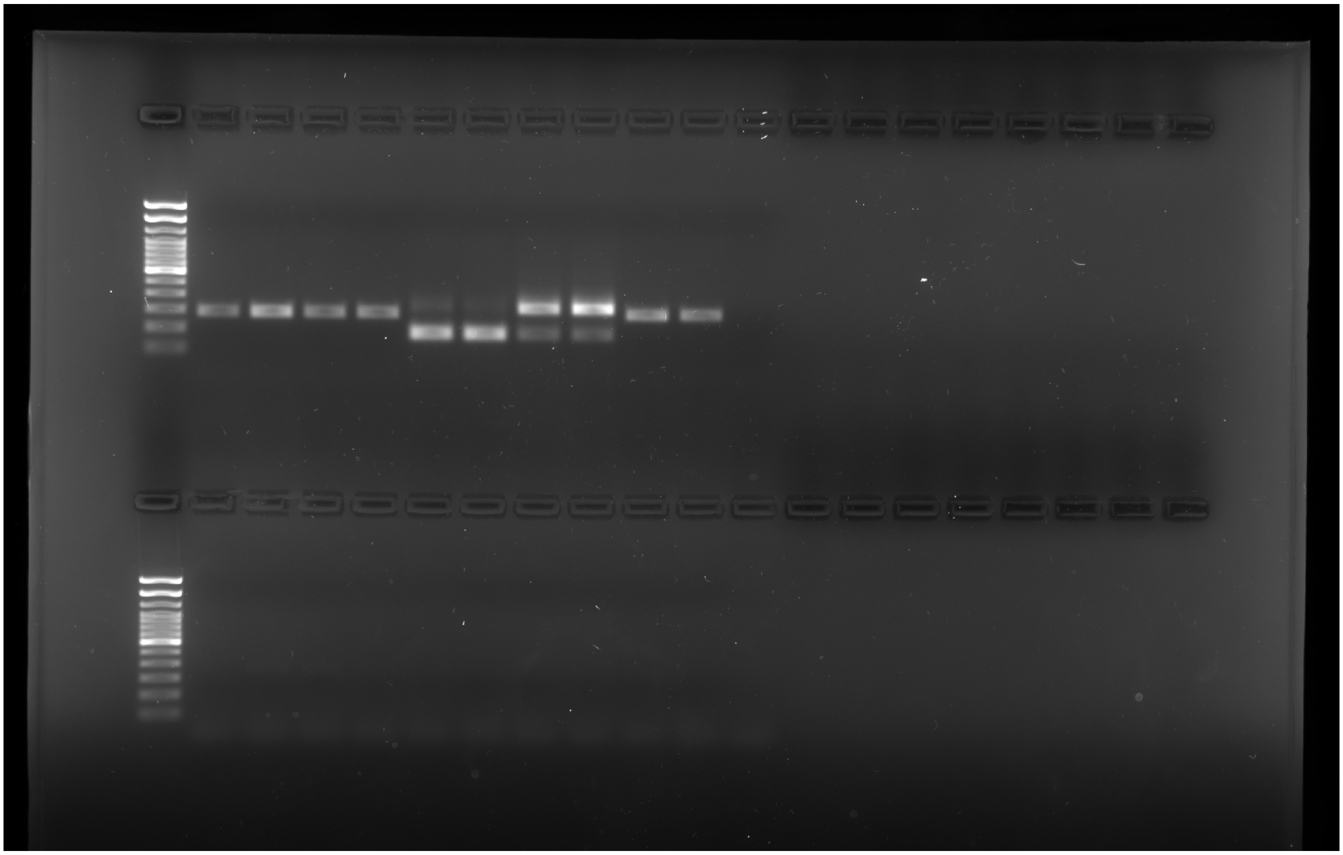

Supplementary Figure S3

Figure 4B uncropped agarose gel picture

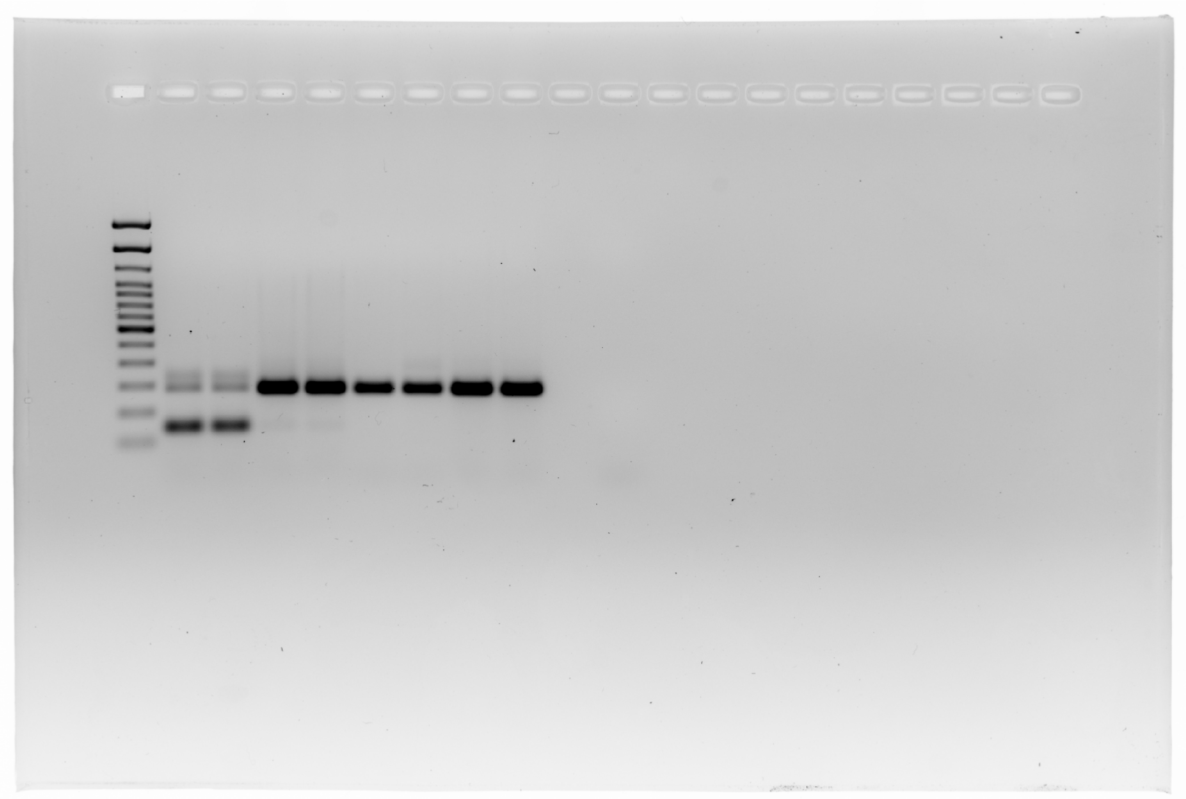

Figure 4C uncropped protein gel picture

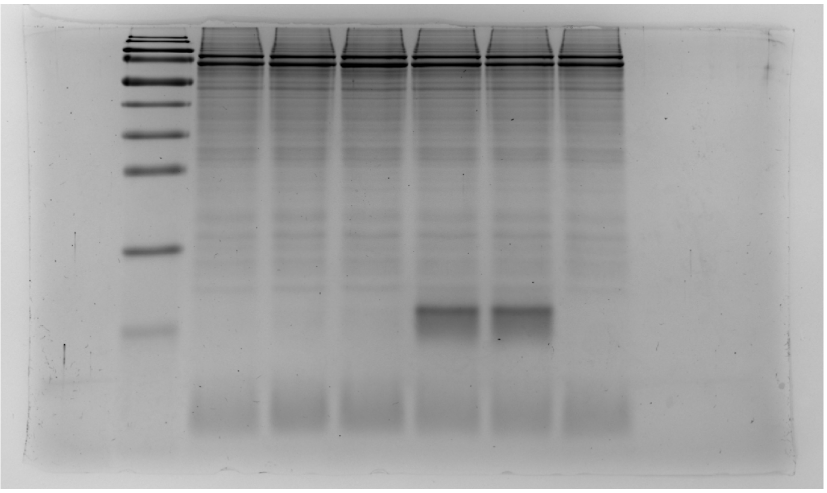

Supplementary Figure S4
